# From Molecular Mechanisms to Clinical Therapy: Understanding Sepsis-Induced Multiple Organ Dysfunction

**DOI:** 10.3390/ijms25147770

**Published:** 2024-07-16

**Authors:** Tijana Srdić, Siniša Đurašević, Iva Lakić, Aleksandra Ružičić, Predrag Vujović, Tanja Jevđović, Tamara Dakić, Jelena Đorđević, Tomislav Tosti, Sofija Glumac, Zoran Todorović, Nebojša Jasnić

**Affiliations:** 1 Faculty of Biology, University of Belgrade, 11000 Belgrade, Serbia; tijana.srdic@bio.bg.ac.rs (T.S.); sine@bio.bg.ac.rs (S.Đ.); djiva@bio.bg.ac.rs (I.L.); a.ruzicic@bio.bg.ac.rs (A.R.); predragv@bio.bg.ac.rs (P.V.); tanja.jevdjovic@bio.bg.ac.rs (T.J.); tamara.dakic@bio.bg.ac.rs (T.D.); jelenadj@bio.bg.ac.rs (J.Đ.); 2 Institute of Chemistry, Technology and Metallurgy, National Institute of the Republic of Serbia, University of Belgrade, 11000 Belgrade, Serbia; tosti@chem.bg.ac.rs; 3 School of Medicine, University of Belgrade, 11129 Belgrade, Serbia; sofijaglumac09@gmail.com (S.G.); zoran.todorovic@med.bg.ac.rs (Z.T.)

**Keywords:** sepsis, multiple organ failure, sepsis treatment, melatonin, metformin, palmitoylethanolamide (PEA), herbal extracts, gut microbiota

## Abstract

Sepsis-induced multiple organ dysfunction arises from the highly complex pathophysiology encompassing the interplay of inflammation, oxidative stress, endothelial dysfunction, mitochondrial damage, cellular energy failure, and dysbiosis. Over the past decades, numerous studies have been dedicated to elucidating the underlying molecular mechanisms of sepsis in order to develop effective treatments. Current research underscores liver and cardiac dysfunction, along with acute lung and kidney injuries, as predominant causes of mortality in sepsis patients. This understanding of sepsis-induced organ failure unveils potential therapeutic targets for sepsis treatment. Various novel therapeutics, including melatonin, metformin, palmitoylethanolamide (PEA), certain herbal extracts, and gut microbiota modulators, have demonstrated efficacy in different sepsis models. In recent years, the research focus has shifted from anti-inflammatory and antioxidative agents to exploring the modulation of energy metabolism and gut microbiota in sepsis. These approaches have shown a significant impact in preventing multiple organ damage and mortality in various animal sepsis models but require further clinical investigation. The accumulation of this knowledge enriches our understanding of sepsis and is anticipated to facilitate the development of effective therapeutic strategies in the future.

## 1. Introduction

Sepsis is a syndrome involving physiological, pathological, and biochemical abnormalities caused by a dysregulated systemic inflammatory response to infection [1,2,3,4]. This complex condition can cause deterioration of multiple inflammatory and oxidative stress parameters, which lead to multiple organ dysfunction, septic shock, and death [2,5]. According to estimates from 2017, there were approximately 48.9 million cases of sepsis worldwide, resulting in 11 million sepsis-related deaths. Consequently, the reported mortality rate was 19.7%, which is a 52.8% decrease compared to the rate in 1990 [6]. However, a meta-analysis of data from Europe and North America between 2005 and 2018 showed that the mortality rate of patients with septic shock was 38% [7].

The primary syndrome and leading cause of death among critically ill sepsis patients is multiple organ dysfunction, estimated to occur annually at an incidence rate of 51%. Multiple organ dysfunction develops as a clinical consequence of various pathophysiological factors, representing an acute and potentially reversible failure of two or more organs [8]. The onset and severity of sepsis-induced organ dysfunction depend on various factors, including patient age, immune system status, comorbidities, and pathogen characteristics such as type, virulence, and site of primary infection [9]. This dysfunction can manifest as vascular dysfunction, cardiomyopathy, acute lung injury (ALI), acute kidney injury (AKI), hepatic failure, and/or dysfunction of the central nervous system [9,10,11,12,13]. Death events are directly associated with the number and type of organ failures. The mortality rate for sepsis patients is below 50% for dysfunction of up to four organs, exceeds 50% for five to seven organs, and reaches 100% for failure of seven or more organs [8].

Despite the significant number of experimental and clinical studies on sepsis, developing effective treatment strategies for critically ill patients, aimed at either preventing organ failure or treating its consequences, remains challenging. This review aims to systematically overview existing treatment methods for sepsis-induced multiple organ dysfunction and explore current research on novel therapeutic approaches.

## 2. Definition of Sepsis

Over time, the definition of sepsis has evolved but still remains heterogeneous and unclear [3,4]. The first recorded mention of sepsis dates from the ancient Greeks, when Hippocrates first proposed the term sepsis (gr. σήψις) for the biological breakdown accompanied by the decay or decomposition of organic matter [14]. Long after that, when inflammation was recognized as redness, swelling, fever, pain, and loss of function, sepsis began to be defined as the body’s response to infection [3,4]. It became clear that it was the host response, not the actual infection, that caused patients to die [3].

Since 1991, sepsis has been defined as a systemic inflammatory response syndrome (SIRS) to infection. SIRS is characterized by at least two symptoms: fever or hypothermia, high heart and respiratory rate, and leukocytosis, leukopenia, or neutrophilia [2,15]. However, it soon became evident that sepsis is a more complicated condition than previously described, requiring a more comprehensive definition. In recent decades, the definition was first changed to an infection complicated by acute organ dysfunction and then, at the Third International Consensus, sepsis was defined as a “life-threatening organ dysfunction caused by a dysregulated host response to infection” whereas septic shock has been described as a subset of sepsis characterized by severe physiological abnormalities significantly elevating mortality risk [2,3]. More specifically, septic shock is characterized by hypotension that persists despite volume resuscitation and requires the use of vasopressors [2,15]. Today, specific clinical criteria are used to identify sepsis. They are united in the Sequential Organ Failure Assessment Score (SOFA), including measures such as respiration, coagulation, bilirubin, and creatinine levels [2]. Of note, the use of quick SOFA score (qSOFA) as the primary screening tool for sepsis has recently been discouraged because there are more sensitive tools like the National Early Warning Score (NEWS) or systemic inflammatory response syndrome (SIRS) score [16].

## 3. Pathophysiology of Sepsis

The cellular and molecular aspects of sepsis pathophysiology are highly complex and involve an imbalanced inflammatory response, dysfunction of the immune system and neuroendocrine-immune network, mitochondrial damage, endothelial dysfunction, coagulation disorders, and autophagy [4,17,18]. Moreover, inflammation can be followed by immunosuppression, which is associated with the inhibition of metabolic processes, including glycolysis, fatty acid oxidation, and oxidative phosphorylation. The intensity of these processes depends on various factors, such as genetics, comorbidities, and pathogen types [17]. This complex host response results in organ dysfunction and often leads to mortality [4].

**Figure 1:**
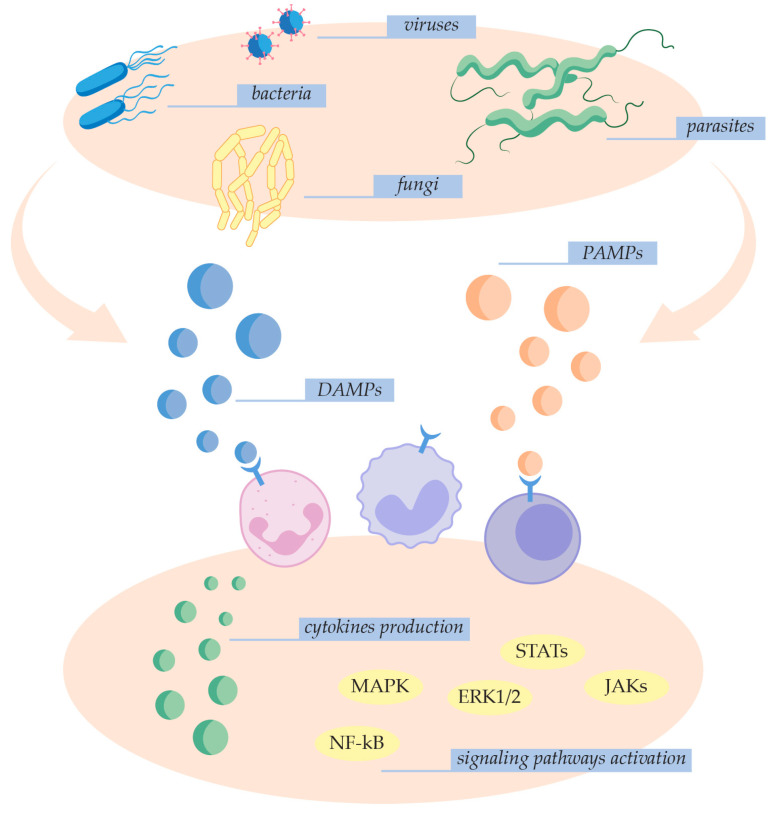
Cascade activation of the immune system in sepsis. DAMPs: damage-associated molecular patterns; PAMPs: pathogen-associated molecular patterns; STATs: signal transducers and activators of transcription; MAPK: mitogen-activated protein kinase; ERK1/2: extracellular signal-regulated kinase 1/2; JAKs: Janus kinases; NF-kB: nuclear factor-kappa B.

Invasive pathogens like bacteria, fungi, parasites, and viruses typically cause activation of the innate immune system consisting of macrophages, monocytes, neutrophils, and natural killer cells [4,17] (Figure 1). Specifically, pattern-recognition receptors (PRRs) recognize and bind pathogen-associated molecular patterns (PAMPs) and damage-associated molecular patterns (DAMPs) [4,19]. PAMPs are exogenous factors originating from infection-derived microbial products like lipopolysaccharide (LPS), flagellin, lipoteichoic acid, and viral RNA and DNA [4,15,20]. DAMPs are endogenous factors such as high-mobility group box-1 (HMGB-1) protein, histones, uric acid crystals, and mitochondrial and genomic DNA released by damaged immune, epithelial, and endothelial cells. Both PAMP and DAMP interactions initiate the activation of neutrophils and specific signaling pathways involved in inflammation, adaptive immunity, and cellular metabolism, including extracellular signal-regulated kinase 1/2 (ERK1/2), mitogen-activated protein kinase (MAPK), Janus kinases (JAKs), signal transducers, and activators of transcription (STATs) and nuclear factor-kappa B (NF-kB) [4,15,17,21]. These events result in the production of inflammatory mediators and lead to the development of pro-inflammatory, pro-apoptotic, pro-adhesive, and pro-coagulant phenotypes [4,22].

Genetic screening in sepsis patients revealed a significant difference in gene expression compared to healthy volunteers [23]. The upregulated genes are included in defensive responses to pathogens, metabolic pathways, complement-dependent cytotoxicity, and apoptosis. Additionally, compared to the non-sepsis group, the expression of MAPK increases gradually from sepsis to septic shock groups [24]. However, downregulated are genes involved in the differentiation of natural killer cells (i.e., natural cytotoxicity triggering receptor 3 (*NCR3*)) and lymphocytes (i.e., CD79a and CD79b molecules (*CD79A* and *CD79B*)), regulation of cytokine production, and antigen-presenting cells (i.e., major histocompatibility complex—class II (*HLA-DQB2*) and phospholipase D family member 4 (*PLD4*)). Future detailed gene expression analyses in sepsis patients can result in the identification of biomarkers for sepsis diagnosis and the prediction of mortality [23].

### 3.1. Inflammation and Oxidative Stress in Sepsis Development

**Figure 2:**
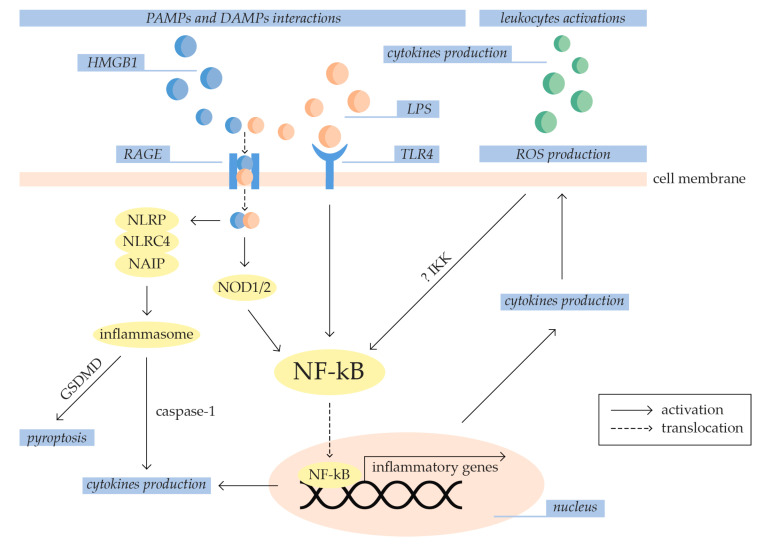
The interplay of inflammation and oxidative stress in sepsis pathogenesis. PAMPs: pathogen-associated molecular patterns; DAMPs: damage-associated molecular patterns; HMGB1: high mobility group box 1; RAGE: receptor for advanced glycation end products; LPS: lipopolysaccharide; TLR4: Toll-like receptor 4; ROS: reactive oxygen species; NLRP: NLR family pyrin domain-containing; NLRC4: NLR family card domain-containing 4; NAIP: NOD-like receptor family apoptosis inhibitory protein; GSDMD: gasdermin D; NOD1/2: nucleotide-binding oligomerization domain-containing protein 1/2; IKK: the inhibitor of NF-kB kinase complex; NF-kB: nuclear factor-kappa B.

The underlying process of sepsis pathogenesis is an imbalanced systemic inflammatory response [4,17]. In inflammation, leukocytes, cytokines, oxygen radicals, endothelial cells, and the complement and coagulation systems are primarily involved. They are an essential part of innate immunity, but their imbalanced activity plays a vital role in developing sepsis [20]. Furthermore, the initial inflammation in sepsis is a critical factor for the propagation of oxidative stress [25]. Oxidative stress occurs when there is an imbalance between the generation of reactive oxygen species (ROS) and the ability of the antioxidant defense system to neutralize them, leading to an increase in oxidant levels [26,27]. ROS production is both a cause and a consequence of inflammation [25,27]. Activated leukocytes release pro-inflammatory cytokines, which increase the expression of ROS-producing enzymes and result in ROS overproduction [4]. Additionally, ROS can initiate the activation of the NF-kB signaling pathway [25,27]. Their production can apparently activate the inhibitor of NF-kB kinase complex (IKK) responsible for NF-kB activation [26]. The following sections will further elucidate the role of inflammation and oxidative stress in the pathogenesis of sepsis, as illustrated in Figure 2.

#### 3.1.1. Inflammation

Toll-like receptors (TLRs) and Nod-like receptors (NLRs) are important PRRs that participate in the initiation of sepsis-related inflammation [19]. The TLRs are a family of membrane-bound proteins with an intracellular Toll-interleukin-1 receptor (TIR) domain. The TIR domain of various TLRs can be activated with different PAMPs including lipoproteins (TLR1/2), peptidoglycan (TLR2), LPS (TLR4), and flagellin (TLR5) [19,28]. Conversely, the NLRs are a large family of cytosolic PRRs involved in inflammasome and non-inflammasome responses to infection [28,29]. Inflammasome-independent NLRs, such as nucleotide-binding oligomerization domain-containing protein 1/2 (NOD1 and NOD2), primarily bind bacterial peptidoglycan derivates, which activate signaling pathways involved in inflammation [19,29]. However, activation of inflammasome-dependent NLRs results in the constitution of the inflammasome, a multimeric protein complex [29,30]. Inflammasome formation can be activated by PAMPs or DAMPs binding to NLR family pyrin domain-containing (NLRP1, NLRP2, NLRP3, NLRP6, NLRP7, NLRP12), NLR family card domain-containing 4 (NLRC4), and NOD-like receptor family apoptosis inhibitory protein (NAIP) [30]. Consequently, inflammasome activates caspase-1, which has an important role in the processing and maturation of pro-inflammatory cytokines [19,29,30,31]. Additionally, it participates in cleaving gasdermin D (GSDMD), which releases its pore-forming domain involved in pyroptosis, a pro-inflammatory and non-apoptotic form of programmed cell death [29,30,31].

Activated TLRs and NODs synergistically induce NF-kB activation through receptor-interacting protein 2 (RIP2), myeloid differentiation protein 88 (MyD88), TIR receptor-inducing interferon-β (TRIF), and various tyrosine kinases [19]. The NF-kB pathway regulates various cellular processes and is vital as a transcription factor in the initial stages of the inflammation process [32,33]. Additionally, NF-kB activation can be induced by a high concentration of extracellular high mobility group box 1 (HMGB1), a most potent DAMP that induces inflammation through massive cytokine production [17]. Specifically, HMGB1 binds to LPS and facilitates its transport through the membrane of vascular endothelial cells and macrophages via the receptor for advanced glycation end products (RAGE) [4]. After activation, NF-kB is translocated to the nucleus, where it binds to promoters of various genes, including tumor necrosis factor (TNF), interleukins (ILs), and interferons (IFNs), resulting in their transcriptional upregulation. This triggers the cascade activation of other pro-inflammatory cytokines [15,32,33]. Cytokines, including interleukins, chemokines, interferons, tumor necrosis factor, and growth factors, are autocrine, paracrine, and endocrine molecules that induce target cell activation, proliferation, or migration. Interleukins, secreted mainly by leukocytes and endothelial cells, are particularly important during infectious processes [19]. However, an imbalance in their pro-inflammatory actions can lead to cell activation, tissue damage, and necrosis [20]. In sepsis, crucial pro-inflammatory cytokines are IL-1β, IL-6, IL-12, and IL-17. IL-1β increases the activity of NF-kB and induces the synthesis of other interleukins, such as IL-8 and IL-1α. IL-12 induces high production of INF-γ, and IL-17 induces the synthesis of additional interleukins, TNF-α, and chemokines by endothelial cells, epithelial cells, fibroblasts, and macrophages. The levels of IL-1β and IL-6 appear to correlate with the severity of sepsis, as they are found at higher levels in deceased patients compared to survivors [19].

#### 3.1.2. Oxidative Stress

Production of ROS is important for normal cellular function, and they have essential roles in cell signaling [34]. However, in oxidative stress conditions, increased ROS production results in their reactions with proteins, carbohydrates, nucleic acids, and unsaturated lipids in cells, leading to cellular damage [26]. In sepsis, the ROS that contributes to disease development includes superoxide (O_2_^•−^), hydrogen peroxide (H_2_O_2_), peroxynitrite (ONOO^−^), hypochlorous acid (HOCl), and the hydroxyl radical (•OH) [26]. Their production increases the activation of the NF-kB pathway, leading to the production of circulating inflammatory mediators such as pro-inflammatory cytokines IL-1β, IL-6, and TNF [25,27]. Additionally, ROS attract the pro-inflammatory chemokines essential for recruiting leukocytes to injured tissue. This initiates the synthesis of IL-1β, IFN, and TNF, indicating further ROS production [25]. Antioxidant defense systems metabolize excessive ROS, employing enzymes such as superoxide dismutase (SOD), glutathione reductase (GR), glutathione peroxidase (GSH-Px), and catalase (CAT) [35]. Manganese-containing SOD (Mn-SOD) converts O_2_^•−^ to H_2_O_2_, which is further processed to H_2_O by CAT or GSH-Px [26]. However, it has been observed that LPS administration alters the imbalance in the antioxidant enzyme ratio. Đurašević et al. (2022) reported a decrease in hepatic CAT and GSH-Px levels but increased SOD levels in LPS-injected rats [36]. Consequently, imbalances in the SOD/CAT ratio, as a consequence of their overexpression or downregulation, have been associated with morbidity in sepsis. Additionally, LPS can upregulate the expression of both ROS-producing enzymes nicotinamide adenine dinucleotide phosphate (NADPH) oxidases (NOXs) and nitric oxide synthases (NOSs) [26]. The NOX enzymes (NOX1-5 and Duox1-2) utilize NADPH as an electron donor to produce O_2_^•−^ from molecular oxygen, which induces other ROS production [37,38]. They have various cellular functions, including differentiation, proliferation, growth, invasion, migration, apoptosis, and immune response. They are also associated with the host–pathogen immunological response and can be induced by DAMPs, PAMPs, and pro-inflammatory cytokines [35]. Conversely, NOS catalyzes the formation of reactive nitrogen species (RNS), like nitric oxide (NO•). Neuronal-NOS (nNOS) and endothelial-NOS (eNOS) are constitutively expressed and have various physiological functions, while inducible NOS (iNOS) is associated with pathological pathways [26]. Sepsis induces de novo synthesis of iNOS, responsible for the NO• overproduction [26,34]. NO• can react with O_2_^•−^ and generate a more reactive ONOO^−^, contributing to ROS overproduction [26,39]. 

The electron transport chain of mitochondria is a central source of intracellular ROS, and mitochondria are a primary site for their reactivity [26,27,34]. Sepsis is associated with morphological changes in mitochondria, including protons leaking through the inner mitochondrial membrane, membrane permeability transition, and potential disruption [25,26]. Mitochondrion dysfunction causes further ROS production and the release of pro-apoptotic proteins, leading to apoptosis. It also disrupts adenosine triphosphate (ATP) synthesis, leading to decreased cell levels of ATP and cellular energy failure, which has been associated with the death of sepsis patients [26,27,40]. Overall, these events reduce oxygen utilization despite normal PO_2_ levels in the peripheral blood, known as cytopathic hypoxia [27,39,40]. These findings are supported by different studies showing decreased PO_2_ in hepatocytes and enterocytes in septic rats. In vitro, it has been observed that cytokines reduce PO_2_ in human enterocytic cells, indicating inflammation involvement in hypoxia development. However, this change was reversible after removing cytokines [39]. Furthermore, sepsis-related inflammation is followed by hypoxia-inducible factors (HIF1-2) activation. The HIF1 and HIF2 complexes are formed during hypoxia as a result of the accumulation of subunits HIF1α and HIF2α in the nucleus. These complexes upregulate the transcription of appropriate genes involved in coagulation, which influences vascular endothelial homeostasis [41].

### 3.2. Sepsis-Related Endothelial Dysfunction

Inflammation and oxidative stress can contribute to endothelial dysfunction, which is integral to sepsis pathogenesis and influences sepsis-induced organ damage [18,20,22]. Walborn et al. (2019) showed a significant correlation between markers of endothelial dysfunction and mortality in sepsis patients [42]. During inflammation, the endothelium actively participates in preventing the spread of pathogens and local infections. However, in sepsis severe endothelial damage contributes to impaired blood flow and tissue perfusion, leading to life-threatening organ damage. Pathogens induce the reprogramming of endothelial cells to a pro-inflammatory phenotype, accompanied by the production of cytokines, chemokines, procoagulants, and pro-adhesive molecules [43]. Sepsis-related endothelial dysfunction is usually followed by damage of endothelial glycocalyx, which is an essential regulator of vessel barrier integrity between the blood and vessel wall, consisting of proteoglycans, glycoproteins, glycolipids, glycosaminoglycans, and adherent plasma proteins. Degradation of its components violates vessel barriers and can expose endothelium to the adhesion of immune cells, which is involved in sepsis-related inflammation [44].

The vascular endothelium is semipermeable, allowing the exchange of fluids and plasma proteins but preventing the penetration of pathogens [18]. In sepsis, pathogens induce inflammatory pathways in endothelial cells through PRRs, leading to NF-kB and MAPK activation [43]. The production of cytokines causes increased expression of adhesion molecules, leading to leukocyte and neutrophil adhesion in vessels, their migration to tissue, and local inflammation [18,44]. Serum levels of adhesion molecules, including E-selectin, P-selectin, intercellular adhesion molecule-1 (ICAM-1), and vascular cell adhesion molecule-1 (VCAM-1), have been observed to correlate with sepsis severity, number of organ failures, and mortality [44]. Furthermore, producing the primary endogenous vasodilator NO• in sepsis also increases endothelial permeability and platelet adhesion [18,43,44]. Cumulatively, these contribute to acute endothelial dysfunction, which induces hypotension, local hypoxia, reduced organ perfusion, and ischemia, leading to organ damage [18].

The vascular endothelium also has a crucial anticoagulant function [18,43]. Therefore, its dysfunction causes coagulation cascade activation, contributing to sepsis-associated coagulopathy. During inflammation, platelet–leukocyte aggregates are formed to promote neutrophil release and prevent the spreading of pathogens. This can cause the aggregates to adhere to the endothelium, resulting in local inflammation [43]. Furthermore, sepsis-associated coagulopathy is characterized by a disbalance in the concentration of various procoagulant and anticoagulant proteins such as thrombomodulin, antithrombin, activated protein C (APC), tissue factor (TF), von Willebrand factor, and plasminogen activator inhibitor 1 (PAI-1) [15,20,32,45]. As a result, the endothelium transitions to a procoagulant state, leading to thrombosis and hemorrhage, loss of endothelial tight junctions, and increased vascular permeability [20,32]. Additionally, thrombocytopenia, a low platelet count, is often seen in sepsis patients along with ongoing microthrombosis. Studies have shown that the severity and outcome of sepsis are closely related to the onset, duration, and degree of thrombocytopenia. This condition might be caused by platelet activation and an imbalance in coagulation factors, leading to the formation of fibrin clots [45]. In sepsis patients, hypoxia is both a consequence and cause of microthrombosis. The formation of small vessel microthrombosis leads to hypoxia and organ damage, while previously described hypoxia due to mitochondrial damage triggers coagulation factors and increases the incidence of thrombosis [41].

Taking into account the aforementioned, some authors have indicated that sepsis often resembles ischemia and reperfusion [46]. In sepsis patients, initial hypoxia and ischemia due to endothelial dysfunction are accompanied by ROS production as a result of mitochondrial damage and the inability to utilize oxygen [22,46]. Moreover, treating sepsis typically involves administering oxygen through mechanical ventilation, which additionally increases ROS production. Therefore, it is crucial to consider administering some mitochondrial redox agents to mitigate oxidative stress effects similar to reperfusion [46].

## 4. Sepsis-Induced Multiple Organ Dysfunction

Sepsis-induced acute failure of two or more organ systems is highly correlated to the mortality of sepsis patients [8,45]. Organ dysfunction ranges from mild damage to complete organ failure and can be repaired unless it contributes to necrotic and gangrenous changes [45,47]. Because of the crosstalk of the systems, dysfunction of a single organ is rare and usually contributes to multiple organ dysfunction [47]. Đurašević et al. (2021, 2022) observed that rats injected with LPS and feces showed signs of systemic inflammation and sepsis effects spreading from one organ to another. They reported that the liver, kidney, and heart of the rats displayed inflammation, oxidative stress, and apoptosis [36,48]. In clinical studies, pathophysiological changes in sepsis patients’ circulatory, respiratory, and nervous systems were observed, including ischemia, electrolyte disturbance, shortness of breath, and a decrease in blood pressure [49].

**Figure 3:**
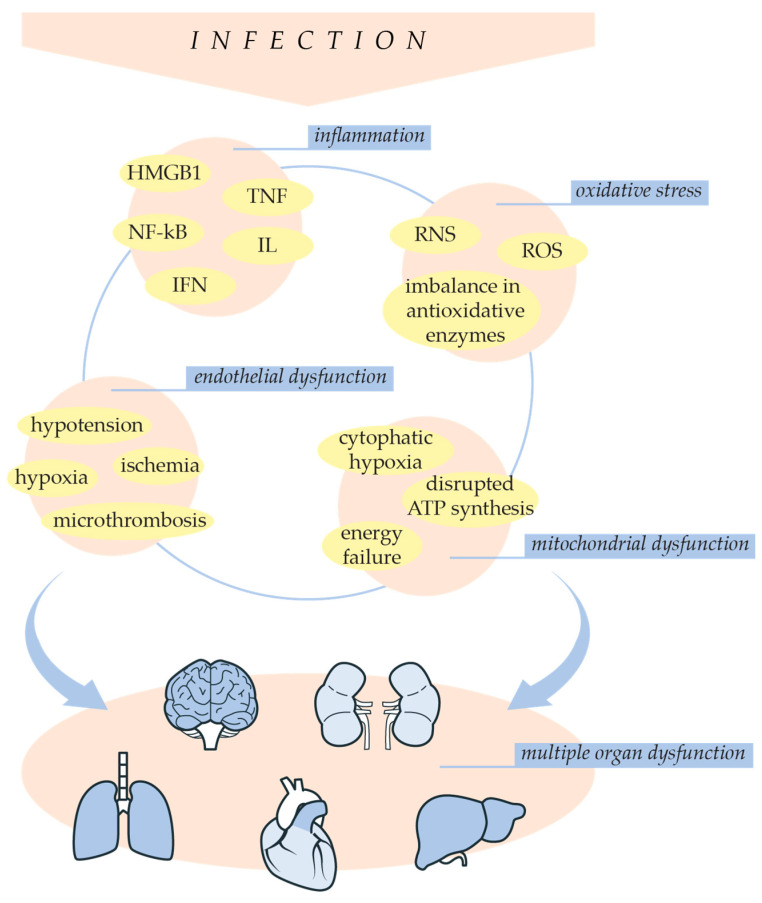
Sepsis-related multiple organ dysfunction. In response to infection, developing inflammation, oxidative stress, endothelial dysfunction, and mitochondrial dysfunction can result in sepsis. Pathophysiological changes are a consequence of the cause–effect link between these processes. Together, they lead to organ damage that can progress to multiple organ dysfunction. HMGB1: high-mobility group box-1; NF-kB: nuclear factor-kappa B; TNF: tumor necrosis factor; IL: interleukins; IFN: interferons; RNS: reactive nitrogen species; ROS: reactive oxygen species.

Multiple organ dysfunction is a complex condition that develops as a consequence of an imbalanced immune response [8]. Sepsis-related infection involves different cause-effect pathogenetic pathways of multiple organ dysfunction (Figure 3) [50]. During sepsis, an inflammatory response results in the uncontrolled and excessive production of cytokines, which leads to a stage known as a cytokine storm. This forms positive feedback between cytokines production and immune cell recruitment. The release of cytokines in the bloodstream contributes to the spread of inflammation and activation of immune cells in organs, leading to organ damage [51]. Cytokines, including IL-6, IL-1β, and TNF-α, induce mitochondrial permeability transition and improve the production of reactive species [50]. Additionally, immune cells, including macrophages, neutrophils, and lymphocytes, produce ROS and RNS [51]. Increased oxidative stress leads to mitochondrial dysfunction, causing cytopathic hypoxia, cellular energy failure, and cell apoptosis, ultimately resulting in organ damage [26,27,50]. In sepsis patients, mitochondrial damage and a decrease in ATP production have been observed in immune cells, the heart, skeletal muscle, and the liver [50]. Structural damage and altered redox state of liver mitochondria were found in severe sepsis patients [34]. Additionally, damage to the mitochondrial membrane results in mitochondrial DNA (mtDNA) leakage in the cytosol. Consequently, mtDNA was observed in the urine of sepsis patients with acute kidney injury (AKI) [52]. These findings suggest that organ failure is enhanced by mitochondrial dysfunction, while studies have shown that improvement in mitochondrial respiration ameliorates sepsis patients’ recovery [34,50]. Furthermore, hypoxia develops due to endothelial dysfunction and reduced tissue perfusion, contributing to sepsis-induced organ dysfunction [18,22]. Severe vasodilation in sepsis patients is followed by blood flow dysregulation between organs, decreased vessel density, and a reduced number of perfused small vessels [39]. In septic shock, endothelial dysfunction is characterized by obstructed blood flow, resulting in microcirculatory abnormalities [22,45]. This can be monitored by signs including skin mottling on the knee area, prolonged capillary refill time (CRT), low tissue oxygen saturation, peripheral ischemia, and oliguria [22]. Vascular microthrombosis decreases oxygen supply to organs, contributing to hypoxia and organ dysfunction [45]. Therefore, endothelial and mitochondrial dysfunction appears to be a fundamental part of sepsis-induced organ failure [18,20,22,34].

### 4.1. Liver Dysfunction

Sepsis-induced liver dysfunction significantly impacts sepsis development and outcome. The mechanism of hepatic damage is related to inflammation and endothelial dysfunction [53]. The liver is an important regulator and a target of the inflammatory response to infection. The LPS increases hepatic cytokine levels, peaking one hour after administration, including IL-1β, IL-6, and TNF-α [54]. This directly activates Kupffer cells to produce ILs, TNF, and IFNs, resulting in the release of acute-phase proteins (APPs) from hepatocytes [9,10]. Fan et al. (2020) also reported that IL-27 is an important cytokine contributing to sepsis-related liver injury. They observed that the release of TNF-α and IL-6 in Kupffer cells can specifically be elevated by IL-27 [53]. Overall, APPs contribute to cytokine production in the liver, accumulating immune cells, and hepatocyte injury [9,10]. This induces APPs and cytokines to be released into the systemic circulation, resulting in widespread inflammation [9]. Therefore, hepatocellular dysfunction can cause complete hepatic failure and severe injury in other organs [10]. Conversely, endothelial dysfunction causes microthrombosis, sinusoidal obstruction, and reduced liver perfusion, leading to hypoxic hepatitis. However, sepsis-induced liver damage can also include cholestasis with unclear etiology. This condition implies impaired bile formation and flow, characterized by increased bilirubin serum levels. Although it has been observed that elevated levels of cytokines and NO• correlate with decreased expression of bile acid transporters, this needs to be further examined [9].

### 4.2. Cardiac Dysfunction

Cardiac dysfunction is associated with increased mortality of sepsis patients by 20% to 50%. It is caused by contractile dysfunction, including biventricular dilatation, decreased ejection fraction, and lower blood pressure, which cannot be improved by intravenous fluid resuscitation and administration of catecholamines [11]. The underlying mechanisms of contractile dysfunction are ischemia, direct myocardial suppression, and mitochondrial dysfunction. As previously mentioned, sepsis-related endothelial dysfunction can alter microcirculation, indirectly contributing to myocardial ischemia [11,55]. However, the direct effect of suppressors on the heart can also alter cardiac function. In sepsis, inflammatory factors such as cytokines and NO• can alter myocardial adrenergic responses, while pathogens, toxins, and DAMPs can directly contribute to cardiomyocyte injury and apoptosis [11]. Busch et al. (2021) showed that IL-1β significantly reduces cardiomyocyte shortening and relaxation speed in the murine CLP model of sepsis. They also reported that IL-1β induces NF-kB activation in cardiomyocytes, promoting inflammatory response in the heart [56]. Furthermore, mitochondrial dysfunction disrupts the synthesis of ATP, which is essential for myocardial contraction [11]. However, in some sepsis patients, myocardial function can undergo downregulation as an adaptive response to low ATP levels. This phenomenon, known as myocardial hibernation, implies short-term cellular dysfunction that resolves with patients’ recovery [55]. However, Kosyakovsky et al. (2021) observed that sepsis is associated with increased long-term risk of myocardial infarction, stroke, and congestive heart failure. This can be explained by inflammatory, endothelial, and coagulation dysregulation contributing to thrombosis [57].

### 4.3. Acute Lung Injury (ALI)

Sepsis patients develop ALI in 25% to 50% of cases, which is characterized by difficulty breathing, cough, fever, and tachycardia. ALI development can increase the sepsis mortality rate by 40%. It is a complex pathogenetic process involving pulmonary vascular endothelial dysfunction, alveolar damage, pulmonary leakage, and fibrosis [12]. In response to infection, inflammatory cell recruitment increases epithelial permeability [58]. Zhou et al. (2019) reported increased levels of IL-6, IL-1β, TNF-α, and IFN-γ in the lungs of LPS-induced ALI in mice [59]. Abnormal activation of macrophages induces overproduction of cytokines, resulting in an uncontrolled inflammatory response and lung damage [12]. Park et al. (2019) described neutrophil recruitment in pulmonary microcirculation of LPS-injected mice. After LPS administration, neutrophils have been demonstrated to be activated and motile, but within 6 h, they gradually accumulate in the capillaries. This can lead to capillary and arteriole obstruction, which forms physiologically dead space in the lung that cannot partake in gas exchange [60]. Furthermore, available oxygen in the lung contributes to tissue damage by producing ROS and damaging mitochondria [12,58]. Collectively, these events cause damage to alveolar walls and increase the permeability of pulmonary vessels [58]. Alveolar epithelial cells are essential in alveolar fluid clearance and pulmonary inflammatory response. Their damage causes leakage of cellular content and violates the lung tissue barrier, contributing to the development of pulmonary edema [12]. In critically ill patients, sepsis-induced ALI can develop into acute respiratory distress syndrome, life-threatening severe lung damage involving hypoxemia, and pulmonary infiltrates [58].

### 4.4. Acute Kidney Injury (AKI)

More than 50% of critically ill sepsis patients develop AKI, which increases mortality risk by 62% [9]. AKI represents a temporary decline of renal function during 7 to 90 days, characterized by decreased glomerular filtration rate, increased serum creatinine, and reduced urine output [9,13,61]. Peerapornratana et al. (2020) observed that the severity and duration of AKI can determine the recovery of sepsis patients or its progression to chronic kidney disease [13]. Furthermore, Pinheiro et al. (2019) reported that sepsis is the main cause of AKI and induces worse outcomes compared to non-sepsis AKI [61]. However, the underlying cause of sepsis-induced AKI is complicated and not fully understood. It can develop due to both pathogenetic processes and sepsis treatment [9]. The pathophysiology of AKI includes inflammation and altered microvascular flow at the peritubular and glomerular levels, leading to reduced renal perfusion, ischemia, oxidative stress, and tubular cell injury [9,62]. Additionally, it has been postulated that AKI develops as an adaptation to metabolic failure, allowing energy distribution. Therefore, organs favor cell survival processes during AKI while inhibiting energy-expensive functions, such as tubular absorption [62]. Janosevic et al. (2021) observed decreased expression of tubular transporters in LPS-treated mice, including sodium-glucose co-transporter-2 (SGLT2). These changes can explain previously reported alterations in water, electrolytes, and glucose homeostasis but also contribute to the adaptation theory. They also reported increased expression of antigen presentation proteins, which can enhance immune response and spread inflammation in the kidney [63]. However, some sepsis treatment protocols can also contribute to AKI. Fluid resuscitation increases volume and renal vascular pressure, causing edema, increased intracapsular pressure, and reduced glomerular filtration rate [9].

## 5. Treatment Strategies for Sepsis

**Figure 4:**
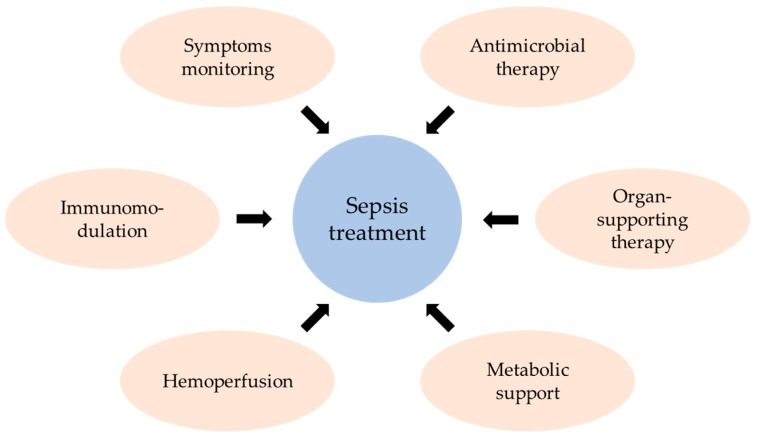
Different strategies are used in the treatment of sepsis.

Sepsis treatment is a complex process involving symptom monitoring, antimicrobial therapy, immunomodulation, hemoperfusion, metabolic support, and organ-supporting therapies (Figure 4) [5,64,65]. Despite various studies, pharmacological treatments for sepsis cannot be clearly defined [65].

### 5.1. Early Diagnosis and Antimicrobial Therapy

Early recognition of sepsis and monitoring of symptoms within the first six hours are crucial for the successful treatment of sepsis [64]. Diagnosing starts with monitoring non-specific physiological characteristics, such as temperature and heart rate, and biomarkers, such as procalcitonin, cytokines (IL-6, IL-10, TNF-α), HMGB1, and coagulation parameters [65]. The early stage of sepsis treatment also involves detecting pathogens and selecting appropriate antibiotic treatment, which can reduce the incidence of sepsis shock by half. Empiric antimicrobial therapy is usually given until the pathogens are identified, mainly through patients’ blood cultures [64,65]. Recently, because this method has low sensitivity to slow-growing microorganisms, it has often been replaced by molecular approaches such as PCR [65]. However, antibiotics can effectively eliminate bacteria in blood plasma but not inside erythrocytes. This is the central issue of antibiotic treatment of sepsis, which reduces the effectiveness of antibacterial and immune therapy. Moreover, the efficacy of this type of sepsis therapy is significantly reduced due to antibiotic resistance among pathogens [64]. Rational use of antibiotics helps minimize side effects, the emergence of bacterial resistance, toxicity, risk of superinfection, and treatment costs [5].

### 5.2. Pharmacological Approaches in Clinical Practice of Sepsis Treatment

Different drugs, as well as their combination, are used to treat sepsis (Table 1). Ibuprofen, a nonsteroidal anti-inflammatory drug, can be used to attenuate the cardiopulmonary response to sepsis, reduce endothelial damage, and decrease inflammation intensity [66]. It also helps reduce fever, tachycardia, and lactic acid levels in sepsis patients but does not significantly affect mortality [67]. Vasoactive drugs like noradrenaline, dopamine, and vasopressin can be used to maintain mean arterial pressure [5,68]. Noradrenaline is more effective than dopamine in septic shock patients since it increases mean arterial pressure in patients with hypotension resistant to fluid resuscitation. Vasopressin is only recommended as a second choice if adequate fluid resuscitation and conventional vasopressors fail to maintain arterial pressure [68]. Additionally, sepsis treatment can involve the use of heparin, thrombomodulin, and antithrombin with the aim of counteracting prothrombotic and procoagulation states, which may be triggered by cytokines like TNF, IL-1, and IL-6, and prevent disseminated intravascular coagulation [64,69,70]. In addition to anticoagulation, heparin has anti-inflammatory and immunomodulatory effects in sepsis, such as modulating platelet activation, leukocyte and neutrophil recruitment, and LPS-induced release of cytokines [71]. Furthermore, statins also alter the coagulation cascade by reducing platelet aggregation and achieve anti-inflammatory and antioxidative effects in sepsis by decreasing leukocyte migration, iNOS and NF-kB activation, and the production of NO•, IL-6, IL-8, and TNF-α [72,73]. In some cases, sepsis patients can receive intravenous immunoglobulin (IVIG) preparations because of decreased IgM and IgG levels, which have been shown to correlate with high mortality rates [74,75]. These preparations can be either monoclonal, developed from a single cell line targeting a specific antigen, or polyclonal, derived from human blood serum and containing different immunoglobulins [75]. IVIG therapy inactivates bacterial endotoxins, promotes phagocytosis, stimulates leukocytes, and modulates cytokine release, which can reduce the mortality of sepsis patients by 34% [74,75].

### 5.3. Metabolic Support

Providing metabolic support is essential for sepsis patients to recover their metabolic condition and reduce morbidity [64]. Metabolic changes in sepsis are complex and include hyperglycemia, insulin resistance, increased protein catabolism, micronutrient decrease, and lipolysis, leading to increased triglycerides but decreased lipoprotein levels [77]. Metabolic support therapy includes monitoring energy requirements, providing nutritional support, and maintaining nitrogen and electrolyte balance [64]. Nutritional support can consist of arginine, glutamine, omega-3 fatty acids, linoleic acid, selenium, and antioxidant supplementation. However, using nutritional support requires careful monitoring of the patient’s individual needs due to their complex responses, which vary depending on the stage of sepsis [78]. Đurašević et al. (2021, 2022) observed the importance of energy production and expenditure in an energy-demanding condition such as sepsis. They reported that meldonium treatment increased mortality in septic rats despite exhibiting anti-inflammatory, antioxidative, and anti-apoptotic effects. It seems that meldonium affected lipid mobilization and altered energy homeostasis, disrupting metabolism and increasing mortality [36,48]. Similarly, ischemia/reperfusion disrupts lipid metabolism by promoting the accumulation of fatty acids, which results in lipotoxicity involving tissue damage and cellular death [79]. Therefore, lipidomics, including the analysis of fatty acid profiles and their dynamic changes, could establish a link between these two conditions and serve as a basis for the development of upcoming clinical strategies [36,48,79].

### 5.4. Septic Shock Treatment

Early initiation of septic shock treatment can delay the onset of sepsis-induced multiple organ dysfunction [5]. It is necessary to start antibiotic therapy within one hour of identifying septic shock, and delaying by an hour increases the risk of mortality by 6% [65]. Septic shock is characterized by tissue hypoperfusion, microcirculation, and cytopathic hypoxia, which result in intense hypovolemia, vasodilation, and cardiac dysfunction [5]. Proper fluid treatment and vasoactive drug therapy can regulate blood volume and maintain tissue function [64]. Furthermore, the treatment of septic shock includes organ-supporting therapies due to mild organ dysfunction [47,64]. This stage of treatment requires a personalized therapeutic approach, including specific organic support, mechanical ventilation, continuous hemofiltration, blood product supply, and nutritional support [5]. While blood purification in hemofiltration is based on a membrane separation process, hemoperfusion involves mass separation by a sorbent [80,81]. Sorbents are molecules with large surface or volume ratios with a high capacity to bind specific compounds from blood by ionic bonds, van der Waals forces, or hydrophobic bonds [80]. It has been shown that direct blood contact with sorbent in an extracorporeal circuit increases clearance compared to hemofiltration. However, due to insufficient clearance of soluble pro-inflammatory mediators, hemoperfusion is only present in renal replacement therapies (RRT) [81]. However, hemoperfusion with an antibiotic polymyxin B can effectively bind endotoxin and interrupt the sepsis development. Consequently, Cruz et al. (2009) showed that adding polymyxin B hemoperfusion to RRT improves organ function and increases the survival of sepsis patients [82].

## 6. Evaluating Sepsis Treatment

Despite advancements in treatment and supportive care, sepsis still has a high mortality rate. In order to effectively treat sepsis, it is crucial that we fully understand its pathophysiology [83]. Sepsis symptoms are not specific, which can lead to delayed diagnosis or inappropriate treatment. Additionally, sepsis patients may have a comorbidity, or the treatment itself may weaken their defense mechanisms [64]. Therefore, various models have been used to research sepsis pathogenesis and drug development.

### 6.1. Experimental Models of Sepsis

Animal models have been crucial in sepsis research, with different approaches including exogenous administration of a toxin or a viable pathogen and compromising the endogenous protective barrier. These models have significantly contributed to our understanding of sepsis pathophysiology and host response to infection [83]. Rodents, particularly mice and rats, have been widely used due to their low cost, ease of breeding, ethical considerations, and similarities to humans in mammal biological characteristics [84]. In addition, genetically modified strains of rodents can be employed to investigate the involvement of specific genes in sepsis pathogenesis [84,85]. However, it is essential to consider that there are still notable differences between rodents and humans in their response to diseases [83,84].

LPS is a toxin widely used to induce sepsis in laboratory rats and mice [83]. It is derived from the membrane of Gram-negative bacteria and triggers infection and systemic inflammation [86]. When LPS is administered intravenously (i.v.) or intraperitoneally (i.p.), it causes acute endotoxemia, resulting in systemic arterial hypotension, lactic acidosis, impaired myocardial contractility, and increased levels of circulating HMGB1, TNF, and IL-6 [85]. However, most laboratory animals are generally less sensitive to the toxic effects of LPS and require a higher dose to display symptoms [83,85]. Injecting bacteria into the peritoneum or blood is another common method used to study the mechanisms of the host response to pathogens in sepsis. However, this method can cause rapid reactions that result in intoxication but not an infection like human sepsis [83]. Furthermore, sepsis can be induced by intraperitoneal injection of feces suspended in saline (FIP). The FIP method is easy to create but has a high mortality rate and does not accurately reflect the development of sepsis in patients [48,87,88].

Cecal ligation and puncture (CLP) and colon ascendens stent peritonitis (CASP) are two commonly used animal models for studying sepsis by altering the endogenous protective barrier [83]. The CLP model mimics human sepsis characteristics by ligating and puncturing the cecum of anesthetized rats or mice and can produce varying outcomes depending on ligation percentage, perforation site, number of punctures, and needle size [86,89]. Conversely, the CASP model involves inserting a stent into the colon ascendens and can be used to mimic surgical interventions in patients as the stent can be removed later [83,84]. Both models cause fecal matter leakage into the peritoneal cavity, resulting in bacterial peritonitis, elevated cytokine release, and multiple organ failure [83,84,86].

### 6.2. Examination of Novel Therapeutical Approaches

Sepsis research aims to improve therapy and develop new treatment approaches, primarily by modulating or interrupting the development of sepsis [64,83]. In sepsis animal models, various drugs have been tested based on their previously demonstrated beneficial effects in different pathological conditions. However, these studies still need to provide more insights into drug effects due to the diverse and variable nature of sepsis pathogenesis. Moreover, some therapeutic agents that can potentially treat sepsis in animal studies have failed in human clinical trials, mainly due to sample heterogeneity, including age variations, genetic background, and comorbidities [83].

Numerous studies explored different therapeutic approaches, such as targeting the mechanism by which bacteria infiltrate erythrocytes, inhibiting PAMPs or DAMPs, and applying anti-inflammatory or antioxidative drugs [17,36,48,64,65]. Bacteria can survive oxidation due to their capsule, layers, or antioxidative defense and penetrate erythrocytes, making them resistant to the immune system and antibiotics [64,65]. Consequently, effective treatment strategies focus on suppressing bacterial antioxidant mechanisms or capsule and biofilm production, eliminating bloodstream bacteria, and developing new antimicrobials [65]. LPS, as a critical PAMP triggering immune system activation, can be another target for sepsis therapy [4,64]. Studies indicate that LPS can be eliminated by binding to low-density lipoproteins (LDLs) and high-density lipoproteins (HDLs). Insulin administration increases LDL and HDL levels, so its application could be a new method in sepsis treatment [64]. Also, one promising approach involves targeting HMGB1, a critical DAMP and a potent mediator of inflammation. Preclinical studies have demonstrated that various therapeutic agents, including active plant components (i.e., glycyrrhizin and naringin) and monoclonal antibodies, can inhibit HMGB1 release from macrophages and improve survival in the CLP model of sepsis. However, further research is necessary to investigate the safety and efficacy of this approach, determine the inhibitors’ bioactivity, identify any potential side effects, and establish appropriate levels of HMGB1 that can prevent damage and promote tissue regeneration [17].

#### 6.2.1. Melatonin

**Figure 5:**
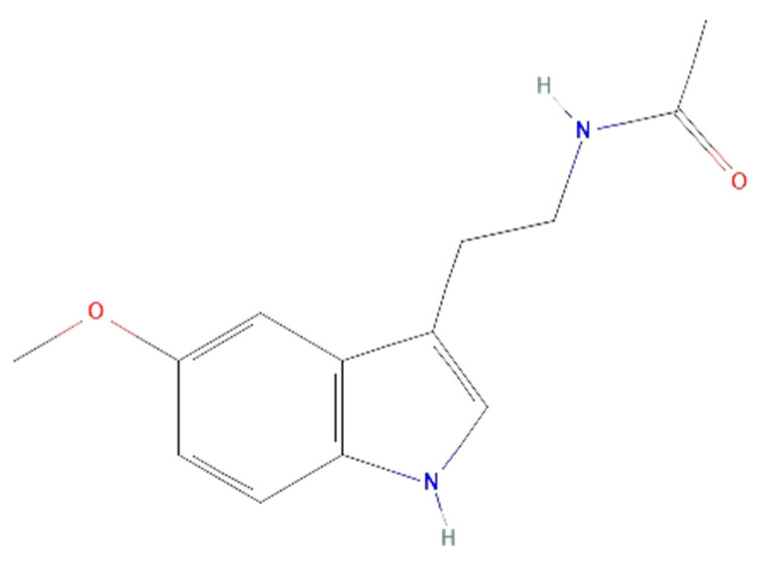
Chemical structure of melatonin [93].

Melatonin (5-methoxy-N-acetyl tryptamine, Figure 5) is a neurohormone mainly secreted by the pineal gland, but it is also synthesized in the retina, gastrointestinal tract, bone marrow, thymus, skin, platelets, cerebellum, and lymphocytes [90,91]. The concentration of melatonin in blood and cerebrospinal fluid has a circadian rhythm since it is secreted in a light/dark cycle, achieving maximum levels during dark hours of the night [90,92]. Melatonin binds to melatonin receptors MT1 and MT2, which cause a decrease in cyclic adenosine monophosphate (cAMP) levels and inhibit various signaling pathways such as ERK1/2. Melatonin can also pass through the membrane and bind to different intracellular receptors [91].

It has been shown that melatonin can achieve various biological effects, such as antiviral, antibacterial, anti-inflammatory, antioxidant, cardioprotective, and neuroprotective [90,94]. Antiviral and antibacterial effects of melatonin have been observed in different preclinical studies of animals infected by various pathogens, including Semliki Forest virus, West Nile virus, *Mycobacterium tuberculosis*, *Chlamydophila pneumonia*, *Staphylococcus aureus*, *Acinetobacter baumannii*, and *Pseudomonas aeruginosa* [90,95]. Anti-inflammatory and antioxidant effects of melatonin include downregulating TLR4, NF-kB, and iNOS expression, inhibiting ROS, RNS, and cytokine production, and upregulating antioxidant systems [91,92]. The cardioprotective effects were demonstrated in the ischemia-reperfusion model, where melatonin pre-treatment significantly reduced infarct size and attenuated subsequent cardiac dysfunction by reducing ROS production and improving antioxidant defense [96,97]. In neurodegenerative diseases with high production of ROS, such as epilepsy, Alzheimer’s, and Parkinson’s disease, melatonin has been shown to prevent neurodegenerative changes and slow disease progression [92].

Considering its beneficial effects, exogenous melatonin administration has been a promising new strategy for treating sepsis [98]. Sewerynek et al. (1995) first showed the protective effects of melatonin in an animal sepsis model. They observed that melatonin reduced LPO in the liver, lungs, and brain and increased levels of GSH-Px in LPS-treated rats [99,100]. Recently, various studies have confirmed that melatonin achieves antioxidative and anti-inflammatory effects in sepsis, mainly by reducing LPO, cytokines, ROS, RNS, and iNOS levels [91,92]. In wild-type and MT1/MT2 double-knockout mice, Fink et al. (2014) observed that melatonin receptors mediate a significant survival increase in sepsis [98]. An improved survival rate was also observed in septic rats, where melatonin decreased HMGB1, IL-1β, and TNF-α levels but increased CAT and SOD production in myocardial tissue [94]. In the CLP rat model, the antioxidative properties of melatonin prevented oxidative organ injury, including liver, kidney, heart, lung, diaphragm, and brain [101]. Melatonin treatment can also alleviate multiple organ injuries by increasing heart rate and decreasing mean arterial pressure, blood glucose levels, and superoxide production in the aorta [102]. It also prevents sepsis-induced renal damage in mice by downregulating IL-1α, IL-1β, and NOX4 expression but increasing the expression of SOD [103]. In the brain of the CLP rat model, melatonin treatment prevented edema, enhanced blood–brain barrier (BBB) function, increased CAT and SOD activity, and reduced levels of NF-kB, HMGB1, IL-1β, and TNF-α [104]. Furthermore, it has been shown that melatonin reduces NO• and iNOS production but increases CAT and SOD activities in the lung and liver, which prevents liver damage and lung edema in CLP-induced sepsis in rats [105]. In ex vivo LPS-treated human leukocytes, melatonin administration reduced membrane potential, ROS and IL-6 production, and oxygen consumption [106]. Furthermore, a recent clinical study showed that co-administration of melatonin with standard sepsis treatment could improve sepsis patients’ outcomes, including SOFA score, the required dose of vasopressor, and the number of ventilator-free and vasopressor-free days [107].

#### 6.2.2. Metformin

**Figure 6:**
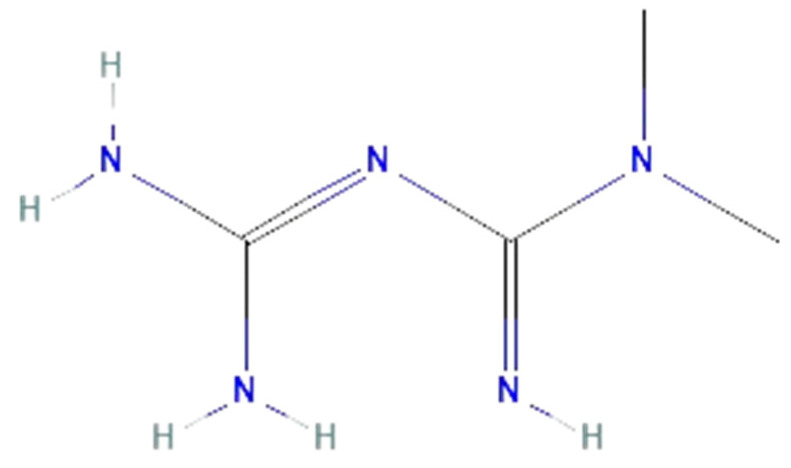
Chemical structure of metformin [115].

Metformin is a synthetic derivate of guanidine, first described in 1968 (Figure 6) [108]. Because of its hypoglycemic activity, metformin has been the primary pharmacological agent for treating type 2 diabetes (T2D) [109,110]. Metformin inhibits the respiratory chain in mitochondria and impacts energy metabolism by inhibiting hepatic gluconeogenesis, reducing intestinal glucose absorption, elevating glucose utilization by peripheral tissues, regulating lipid homeostasis, and improving insulin sensitivity [109,111,112]. The protective effects of metformin are based on the activation of adenosine monophosphate kinase (AMPK) and inhibition of NF-kB pathways [111,113]. AMPK activation maintains energy homeostasis by redirecting the ATP-consuming processes towards ATP-producing catabolic pathways, which reduces lipid stores and enhances insulin sensitivity [114]. In macrophages, metformin reduces the NAD/NADH ratio, production of ROS, NO•, IL-6, IL-1β, and TNF-α, glycolytic and tricarboxylic cycle intermediates, and phosphates levels [110,113]. Additionally, the cardioprotective effects of metformin were shown in endothelial dysfunction, myocardial infarction, acute myocarditis, and chronic heart failure [109]. In addition to its antioxidative and anti-inflammatory effects, metformin improves cardiovascular risk profile, including hemoglobin and cholesterol levels, body mass index, atherogenic dyslipidemia, blood pressure, procoagulant state, and thrombosis [109,113]. Furthermore, metformin exerted protective effects in chronic kidney disease, diabetic nephropathy, cerebral ischemia, Alzheimer’s and Parkinson’s disease, multiple sclerosis, and motor neuron diseases [109].

Recently shown antioxidative and anti-inflammatory effects of metformin position it as a potential candidate for sepsis treatment [111]. In septic patients with T2D, a reduced mortality risk due to preadmission of metformin was observed [116,117]. In preclinical studies, metformin administration improved the survival of septic animals and attenuated brain, lung, heart, liver, and colon injury [118,119,120,121,122]. In the brain of the CLP mice model, metformin reduced the production of IL-6, IL-1β, and TNF-α, prevented LPO, and increased SOD activity, which prevented cerebral edema and BBB damage [118]. Sepsis-induced liver injury was also attenuated in metformin treatment by reducing levels of cytokines, HMGB1, and MAPK but upregulating AMPK expression [123]. Recent studies of the CLP rat model have shown that metformin can prevent inflammation, edema, and injury in the brain, lungs, liver, and colon by regulating gut microbiota [122,124,125]. However, those findings need to be further investigated. Additionally, downregulation of TLR4 expression was observed after in vitro metformin administration in LPS-induced vascular smooth muscle cells (VSMCs) isolated from rats’ aorta [126]. The beneficial effects of metformin were also shown in macrophages and epithelial cells in vitro [119,127]. In LPS-induced mouse macrophages, metformin decreased cytokine production and expression of iNOS and HMGB1 [119]. Furthermore, metformin administration in LPS-induced human bronchial epithelial cells increased cell viability, reduced IL-6 and TNF-α levels, and suppressed NF-kB activation [127].

#### 6.2.3. Palmitoylethanolamide (PEA)

**Figure 7:**
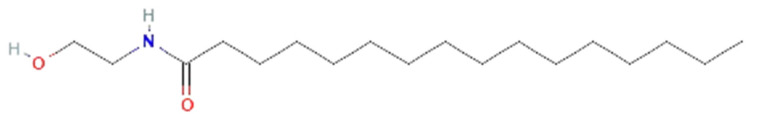
Chemical structure of palmitoylethanolamide (PEA) [128].

Palmitoylethanolamide (PEA) is a highly lipophilic compound and one of the most common N-acyl ethanolamines (Figure 7) [128]. It can be found in mammalian tissues and various food sources such as soybeans, common beans, garden peas, tomatoes, corn, soy lecithin, and peanuts [129].

It has been observed that PEA can achieve anti-inflammatory, antioxidant, antibacterial, anticoagulation, neuroprotective, analgesic, and antinociceptive properties [129,130,131,132,133,134]. The primary receptor of PEA is peroxisome proliferator-activated receptor alpha (PPAR-α). It can also act via transient receptor potential vanilloid 1 (TRPV1), orphan receptor GPR55, and cannabinoid receptors CB1 and CB2 [128,129]. PPAR-α is a nuclear receptor and transcription factor that regulates gene expression in various tissues, including the heart, liver, kidney, muscle, adipose tissue, and immune cells [129]. Activated PPAR-α forms a complex with the retinoic acid receptor (RXR), which translocates to the nucleus and binds to a peroxisome proliferator response element of genes involved in fatty acid transport and metabolism, as well as inflammation and oxidative stress [128,129]. PEA can also activate GPR55 in the gastrointestinal tract and brain, CB1 in presynaptic terminals, and CB2 in monocytes, macrophages, lymphocytes, spleen, tonsils, and thymus gland [129].

Berdyshev et al. (1998) conducted one of the first studies on PEA effects in the sepsis model. They showed that PEA reduces TNF-α production in LPS-induced bronchopulmonary inflammation in mice but does not inhibit neutrophil recruitment [135]. A more recent study also showed that PEA reduces the secretion of TNF-α in LPS-induced adipocytes and LPS-treated mice [136]. Administration of PEA, as a pretreatment or poststimulation, increased cell viability in the LPS-treated co-culture system of human airway epithelial cells and monocytes. This study also showed reduced expression of TLR4, production of ROS and NO•, and levels of IL-6, IL-1β, and TNF-α in PEA-treated groups [137]. Neuroprotective effects of PEA were shown in vitro in LPS-treated mice microglial cells and in vivo in septic rats [132,133]. In LPS-stimulated mice microglial cells, PEA administration reduced iNOS expression and IL-6, IL-1β, and TNF-α levels, which preserved the density, cell area, and perimeter of culture [132]. PEA pretreatment of septic rats prevented LPS-induced brain injury by downregulating NF-kB and iNOS [133]. Furthermore, in LPS-treated rats, PEA also alleviates coagulopathy and inflammation in the lungs by altering blood coagulation parameters, including platelet count reduction and prothrombin time increase, as well as lowering the production of pro-inflammatory mediators NF-kB, IL-6, IL-1β, TNF-α, and IFN-γ [134]. Additionally, it seems that PEA can mitigate the effects of Escherichia coli infection [130,131]. Redlich et al. (2014) observed that phagocytosis of *E. coli* is increased in PEA-stimulated macrophages compared to unstimulated ones. They also showed that after intraperitoneal and intracerebral administration of *E. coli* in mice, PEA reduces *E. coli* concentration in blood and spleen and cytokine production [130]. Moreover, it has been shown that in mice with *E. coli* meningoencephalitis, PEA pretreatment can improve survival rates by reducing *E. coli* concentration in blood, spleen, and liver as well as the release of cytokines and chemokines [131]. In addition, preclinical studies demonstrating the potential of PEA treatment in LPS- and *E. coli*-induced sepsis, it is necessary to investigate its effects on numerous other parameters.

#### 6.2.4. Herbal Extracts

Different herbal extracts can have beneficial effects on sepsis, including antioxidative, anti-inflammatory, immunomodulatory, hematological, neuroprotective, and cardioprotective effects, as well as prevent gastrointestinal barrier disruption and organ failure [138]. Table 2 shows active components present in different plants whose protective effects have been observed in various models of sepsis.

*Aloe vera.* Leaf extracts of *Aloe vera* contain polyphenolic anthraquinones—aloin, aloe-emodin, and rhein, which were previously shown to have antitumor, antibacterial, antiviral, antioxidant, hepatoprotective, anti-inflammatory, and immunomodulatory effects [140,141]. In sepsis, their protective effects have been demonstrated in LPS and CLP models [139,140,141]. *Aloe vera* metabolites decreased iNOS expression, production of NO•, IL-6, and TNF-α, and activation of ERK1/2 but increased SOD and GSH-Px activity in LPS-treated mice [140,141]. Moreover, in the CLP model, *Aloe vera* extracts significantly decreased the mortality of treated mice [139].

*Coleus forskohlii*. Forskolin (FSK) and isoforskolin (ISOF) are labdane diterpenes from the root of the Asian plant *Coleus forskohlii* [180,181]. FSK and ISOF activate adenylyl cyclases, increasing intracellular cAMP, which results in bronchodilatation, vasodilatation, hypotension, secretion of insulin and thyroid hormones, lipolysis, and inhibition of platelet activation and histamine release [181]. Studies have shown their beneficial effects on different pathological conditions such as cystic fibrosis, asthma, obesity, cardiovascular diseases, cancer, diabetes, glaucoma, and liver fibrosis [180,182]. ISOF pretreatment has been shown to prevent acute lung injury development and decrease the mortality rate of LPS-treated rats [145]. FSK and ISOF pretreatment also reduce TLR4, NF-kB, IL-1β, IL-6, and TNF-α levels of LPS-treated human mononuclear leukocytes [146]. However, the potential use of those diterpenoids in sepsis treatment requires further examination.

*Curcuma longa.* Curcumin, a component in the *Curcuma longa* rhizome, has anti-inflammatory, antioxidant, antibacterial, antiviral, and antifungal properties that potentially can be used in sepsis treatment [149]. In an in vitro study on LPS-treated human macrophages, curcumin reduced IL-6 and TNF-α levels and ERK1/2 and STAT1 activation [150]. Animal sepsis model studies have shown that curcumin can block TLR4, decrease ROS production, cytokines levels, and iNOS and NF-kB expression, but elevate antioxidative enzyme levels such as SOD, which reduces multiple organ failure and mortality rates [150,151,152].

*Glycyrrhiza glabra*. Root extract of *Glycyrrhiza glabra* contains triterpene glycoside glycyrrhizin, which has hepatoprotective, anti-inflammatory, and antiviral effects [155]. Recent studies reported that glycyrrhizin can also prevent mortality in rats with sepsis by attenuating inflammation and multiple organ dysfunction [154]. In the CLP model, glycyrrhizin reduced HMGB1, IL-1β, IL-6, and TNF-α on protein and mRNA levels in rats and mice [154,155]. However, complete insights into the mechanisms of glycyrrhizin’s effects on survival in sepsis demand further investigation.

*Panax ginseng.* Ginsenosides are a class of several active components found in the root and rhizome of the Asian plant *Panax ginseng* [157]. They have previously shown immunomodulatory, neuromodulatory, antithrombotic, antibacterial, antiviral, and antitumor effects [157,159]. Different in vitro and in vivo studies have shown the beneficial effects of ginsenosides in sepsis. In septic mice, ginsenosides inhibited ROS production, decreased IL-1β and TNF-α levels, and downregulated TLR4 expression. Treatment with ginsenosides has also reduced HMGB1 release in LPS-treated human endothelial cells and CLP mice models [157]. Additionally, ginsenosides have been shown to prevent organ damage such as heart, liver, lung, and kidneys by reducing NO• and cytokines levels and TLR4 and NF-kB expression [157,158].

*Piper nigrum. Piper nigrum* has been known for improving digestion and treating fever, but it also has antimicrobial, antioxidant, anticancer, and hepatoprotective effects. The effects of its biologically active components, piperine and pellitorine, have been studied in sepsis models [149]. In LPS-induced sepsis in mice, piperine has shown protective impacts, such as inhibiting IL-1β, IL-6, and TNF-α production, STAT1 activation, and IFN release, as well as neutrophil infiltration and lung edema [162,163]. It has been observed that pellitorine treatment reduces HMGB1 release in CLP model and LPS-treated mice. Additionally, an in vitro study has shown that pellitorine not only inhibits the release of HMGB1, which decreases cytokine levels but also reduces vascular permeability and migration of leukocytes to the endothelium [161]. These findings suggest that piperine and pellitorine can potentially be used in sepsis treatment.

*Rhodiola rosea*. Salidroside, a phenylpropanoid glycoside found in the root of *Rhodiola rosea*, has various pharmacological effects such as anti-aging, anticancer, anti-inflammation, antivirus, antioxidative, and hepatoprotective [166,167]. The potential protective effect of salidroside has also been shown in different sepsis animal models. An in vitro study on LPS-treated macrophages showed that salidroside treatment reduces HMGB1 and iNOS production [168]. Furthermore, it has been demonstrated that salidroside also suppresses NO•, TNF-α, IL-6, and IL-1β production [167,168]. The same result was observed in the CLP model in mice, as well as reduced iNOS expression and NF-kB activation, which prevented acute lung injury and decreased mortality rate [166,168].

*Syzygium aromaticum.* Eugenol and biflorin are the primary compounds found in the *Syzygium aromaticum* extract [170]. Their antimicrobial, antinociceptive, antioxidant, anti-inflammatory, and antitumor properties are well-characterized [149,170]. Furthermore, their anti-inflammatory and antioxidant effects were observed in LPS-treated septic mice [170,171,172]. Biflorin has been shown to reduce iNOS expression, NO• production, levels of IL-6 and TNF-α, and STAT1 activation [148]. Eugenol also decreases the production of cytokines such as TNF-α, IL-6, and IL-1β, as well as NOXs activity and NF-kB expression. However, the effect of eugenol on the activity of antioxidative enzymes, including SOD, CAT, and GSH-Px, remains unclear. Different studies on acute lung injury in sepsis showed that eugenol could either decrease or increase their activity, which needs further investigation [171,172].

*Zingiber officinale.* Rhizome of *Zingiber officinale* contains the phenolic alkanones 6-gingerol and zingerone with various pharmacological properties such as antioxidant, anti-inflammatory, antimicrobial, antithrombotic, antitumor, neuroprotective, and cardioprotective [149,175,176,177]. It has been reported that both 6-gingerol and zingerone achieve anti-inflammatory and antioxidant effects in LPS-induced and CLP model sepsis [175,176,177]. It has been observed that zingerone reduces cytokines levels, including TNF-α, IL-6, IL-1β, and IL-10, as well as ROS production, which decreased the mortality rate of septic mice and rats [175,177]. In the CLP mice model, 6-gingerol also increased the survival of treated mice by reducing levels of HMGB1 and IL-1β [176].

#### 6.2.5. Modulating Gut Microbiota

The gastrointestinal tract is the largest surface exposed to the external environment, which is why the immune system must develop a well-balanced tolerance to commensal bacteria while simultaneously offering protection against pathogens [183]. The mucus layer is one of the vital protective mechanisms with various mechanical, chemical, and biological roles. It forms a coat over intestinal cells, increasing in density from the proximal to the distal gut. The mucus layer protects the epithelial cells from external and toxic substances, digestive enzymes, and bacteria, but it also binds water, preventing dehydration and mechanical stress [184]. Moreover, the mucus layer collaborates with the immune system by reducing antigens and pathogens exposure to the immune defense compartments of the enterocytes [183,184]. The gut microbiota is an integral part of the gastrointestinal tract, which contains a spectrum of bacteria, viruses, fungi, parasites, and archaea [185]. It interacts with mucus, which provides nutrients and attachment sites for different microorganisms. Microbial density also increases from the proximal to the distal gut, as well as from the epithelial cells to the lumen. The gut microbiota can influence the formation of the mucus layer, and different microorganisms and their metabolites, including LPS, may play a role in this process. Therefore, it was observed that germ-free mice have a thinner mucus layer that is penetrable to bacteria [184].

The gut microbiota is the primary source of LPS that can be tolerated. Different bacteria produce various LPS types, which differentially influence the immune system. In healthy individuals, the LPS produced by commensal bacteria does not activate TLR4 in intestinal cells. However, modulating the gut microbiota can cause the intestinal cells’ inflammatory response to LPS [186]. Additionally, disrupting the gut mucus and epithelial layers causes LPS penetration into the systemic circulation, which activates macrophage migration and production of IL-1, IL-6, IL-8, IL-10, and TNF-α, resulting in local inflammation [185,186]. Various factors can modulate the gut microbiota, including age, sex, diet type, antibiotic use, and host genotype [185]. However, modulating microbiota can sometimes lead to dysbiosis, an altered state of the microbial community from its standard structure [187]. Studies have shown that consuming meat- and plant-dominant diets can lead to significantly different gut microbiomes [185]. Meals rich in fat and milk can increase LPS levels in the gut, while chronically lower LPS levels are found in consumers of meals rich in fruit and vegetables. However, it is not clarified if some foods contain LPS, induce its production in the gut, or modulate gut microbiota, which facilitates LPS penetration [186]. The use of antibiotics can also cause dysbiosis, which can lead to various infections and metabolic disorders [185]. Antibiotic treatment decreases diversity and eliminates commensal bacteria, while probiotics can suppress LPS-associated inflammation [185,188].

Recent studies underscore dysbiosis as a significant sepsis risk factor [187,188,189]. Genome analysis of gut microbiota in sepsis patients showed that its disruption is linked to sepsis-induced organ damage [188,189]. The intestinal pathogens reach the liver through the portal vein, while disruption of the immune system in sepsis can cause failure in their clearance and allow the spread of infection [189]. Conversely, sepsis-related liver dysfunction induces cytokine production, contributing to mucus barrier disruption and dysbiosis [10]. Therefore, gut–liver crosstalk appears critical in developing sepsis and can be crucial in its treatment. Furthermore, dramatic microbiota alterations correlate with sepsis progression and critical stages [188]. Clinical studies on critically ill sepsis patients have indicated that gut microbiota could be informative for predicting sepsis risk, progression, and death possibility [190,191,192]. They describe two specific dysbiosis patterns, with one present in more severe septic shock patients [191]. They also found dramatic changes in gut microbiome diversity during intensive care of patients, corresponding to different severity of sepsis [192]. Accordingly, septic shock patients have a low diversity of gut microbiota, with individualization of a single genus [189]. Therefore, it seems that the gut microbiota can be a prognostic sepsis marker but also a target for its therapy [188,189].

Probiotic treatment has been shown to modulate the gut microbiome composition, improve gut barrier and immune defense, and prevent pathogen invasion and metabolic disorders. Additionally, probiotics can be co-administrated with prebiotics, known as synbiotic therapy. Prebiotics are non-digestible nutrients that promote the growth of commensal bacteria in the gut, restoring gut microbiome composition and preventing LPS penetration [188]. However, probiotic efficiency varies due to its formulations, while other methods provide many bacterial species [187]. One such method is fecal microbiota transplantation (FMT), which implies the administration of healthy donor feces. This procedure can restore gut microbiota composition, suppress inflammatory reactions, and prevent pathogen penetration [188]. In sepsis, FMT increases commensal bacteria and pathogen clearance, improving the immune system and increasing survival. However, the FMT method must be improved and standardized, including adjustments of donor selection and fecal suspension preparation and transplantation [188,189]. Another novel strategy in modulating gut microbiota is selective digestive decontamination (SDD). SDD implies using specific antibiotics that suppress pathogens but not commensal bacteria due to their resistance. However, a few studies have shown different SDD effects, so this type of therapy requires further research [187,189]. In the future, adaptations of molecular protocols for analyzing and modulating gut microbiomes can potentially be an essential part of sepsis treatment [190].

## 7. Conclusions and Future Perspective

Considering the aforementioned points, sepsis is evidently a multifaceted systemic disorder. Despite significant advancements in treatment over the past three decades, the mortality rate remains alarmingly high. Consequently, further investigation into the mechanisms underlying sepsis-induced mortality is imperative to devise novel therapeutic strategies. The present review is crafted with this objective, offering an extensive overview of the contemporary understanding of sepsis’s molecular and physiological mechanisms alongside conventional and experimental treatment modalities.

Exploring agents influencing metabolic modulation is of particular significance, given recent insights suggesting the pivotal role of energy substrate utilization in effective recovery management. Furthermore, the growing body of research over the past decade underscores the critical role of microbiota, not only in sepsis but also in various inflammatory processes and numerous other pathological conditions. This expanding area of investigation holds promise for developing modern preventive and therapeutic approaches to numerous diseases.

Thus, we anticipate that this review will serve as a valuable resource for researchers endeavoring to elucidate the pathophysiological mechanisms underlying sepsis and related disorders, such as ischemia/reperfusion injury. We hope that such insights will catalyze the emergence of innovative ideas in both experimental and therapeutic arenas, ultimately advancing the management of these conditions and safeguarding human health.

## Figures and Tables

**Table 1:** Overview of different pharmacological approaches in clinical practice of sepsis treatment.

Drug	Dose	Effects	References
Ibuprofen	10 mg per kg	reduces fever, tachycardia, and lactic acid levels; attenuates cardiopulmonary response; decreases endothelial damage and inflammation	[66,67]
Noradrenaline	5 μg per kg per minute	increases arterial pressure	[5,68]
Dopamine	5–15 μg per kg per minute	increases arterial pressure	[5,68]
Vasopressin	0.01–0.04 units per minute	increases arterial pressure	[5,68]
Heparin	10,000–15,000 units per day	anticoagulation; modulates platelet activation, leukocyte and neutrophil recruitment, and LPS-induced release of cytokines	[64,71]
Thrombomodulin	0.06 mg per kg per day	anticoagulation	[69]
Antithrombin	6000 units per day	anticoagulation	[70]
Statins	10–40 mg per day (pravastatin, rosuvastatin), 10–80 mg per day (simvastatin, atorvastatin), 20–80 mg per day (lovastatin, fluvastatin)	reduces platelet aggregation and leukocytes migration; decreases NO•, iNOS, NF-kB, and cytokines levels	[72,73]
IVIG	7 mL per kg per day (Pentaglobin, Biotest Pharma), 0.6 g per kg per day (Polyglobin, Bayer Biological Products), 0.4 g per kg per day (Sandoglobulin, Sandoz Pharmaceutical Corp)	inactivates bacterial endotoxins; promotes phagocytosis; stimulates leukocytes; modulates cytokines release	[74,75,76]

IVIG—intravenous immunoglobulin; NO•—nitric oxide; iNOS—inducible nitric oxide synthases; NF-kB—nuclear factor-kappa B.

**Table 2:** Active components from different plant species and their effects on sepsis.

Plant Species	Active Component	Chemical Structure	Effects	References
*Aloe vera*	aloin	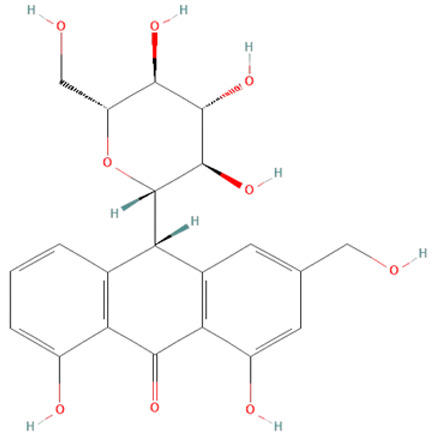	decrease iNOS, NO•, IL-6, and TNF-α production, and ERK1/2 activation; increase SOD and GSH-Px activity	[139,140,141,142,143,144]
	aloe-emodin	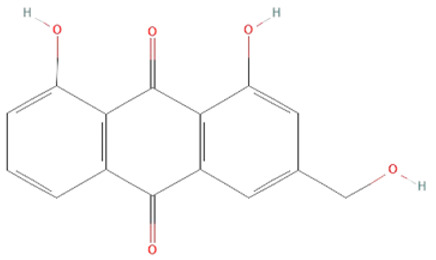		
	rhein	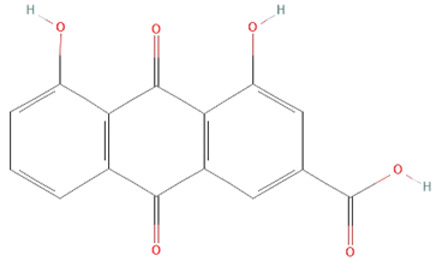		
*Coleus forskohlii*	forskolin	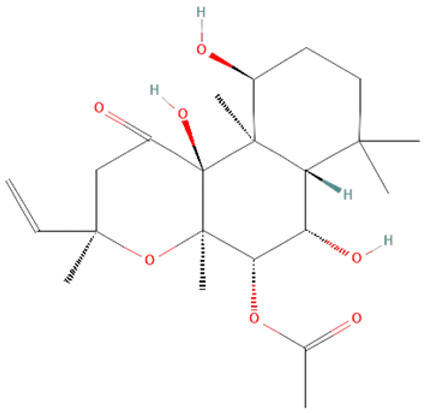	increase cAMP levels, decrease TLR4, NF-kB, IL-1β, IL-6, and TNF-α levels	[145,146,147,148]
	isoforskolin	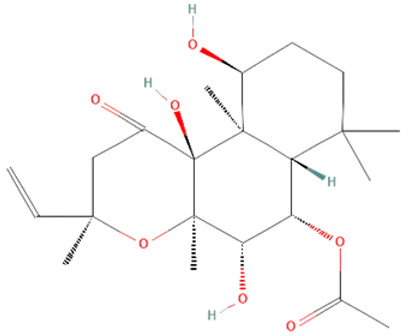		
*Curcuma longa*	curcumin	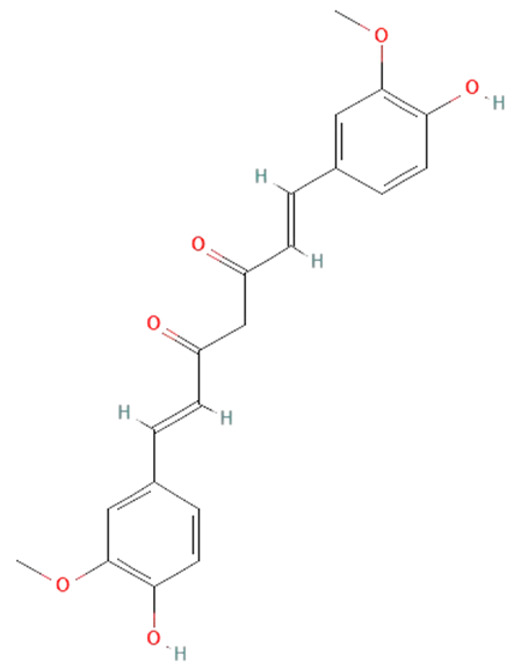	inhibits TLR4, NF-kB, iNOS, STAT1, and ERK1/2 activation; reduces ROS and cytokines levels; increases SOD levels	[149,150,151,152,153]
*Glycyrrhiza glabra*	glycyrrhizin	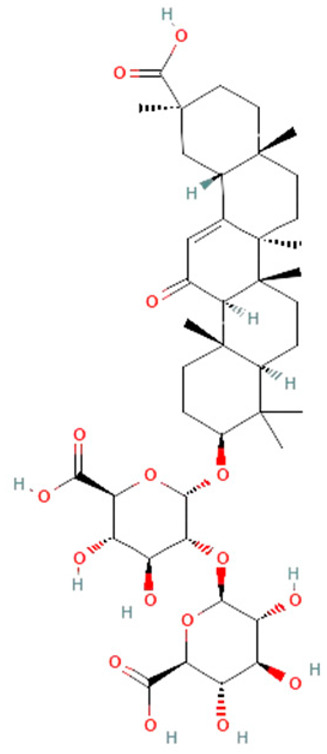	reduces HMGB1, IL-1β, IL-6, and TNF-α	[154,155,156]
*Panax ginseng*	ginsenosides	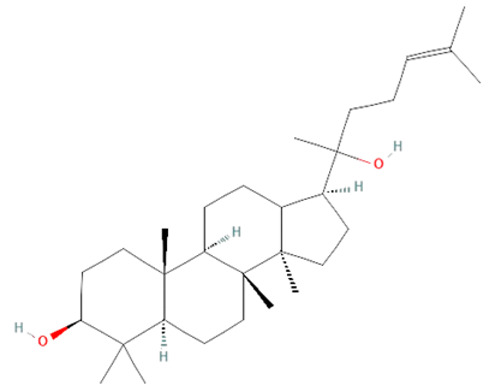	reduce ROS, HMGB1, and cytokines levels; inhibit TLR4 and NF-kB expression	[157,158,159,160]
*Piper nigrum*	piperine	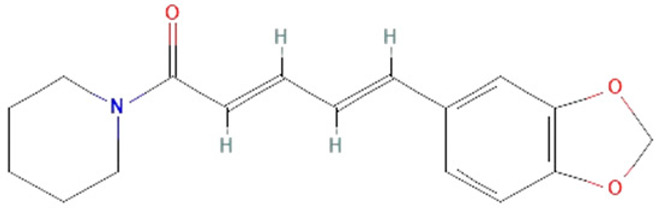	decrease HMGB1, IL-1β, IL-6, TNF-α, and IFN levels, STAT1 activation, neutrophil infiltration, vascular permeability, and migration of leukocytes to the endothelium	[149,161,162,163,164,165]
	pellitorine	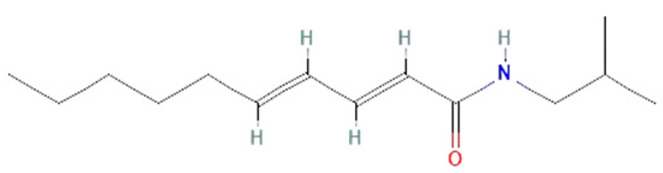		
*Rhodiola rosea*	salidroside	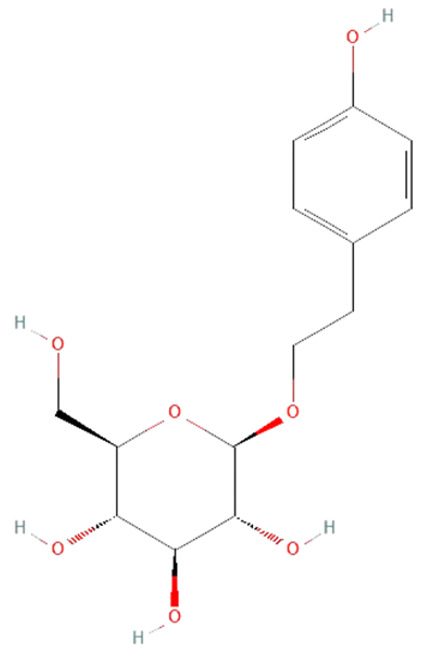	reduces HMGB1, iNOS, NO•, TNF-α, IL-6, and IL-1β production, and NF-kB activation	[166,167,168,169]
*Syzygium aromaticum*	eugenol	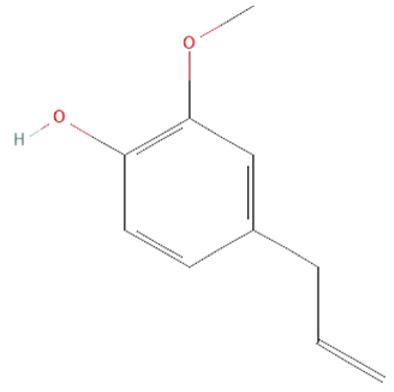	reduce levels of STAT1, NF-kB, NOXs, iNOS, NO•, IL-6, TNF-α, and IL-1β	[149,170,171,172,173,174]
	biflorin	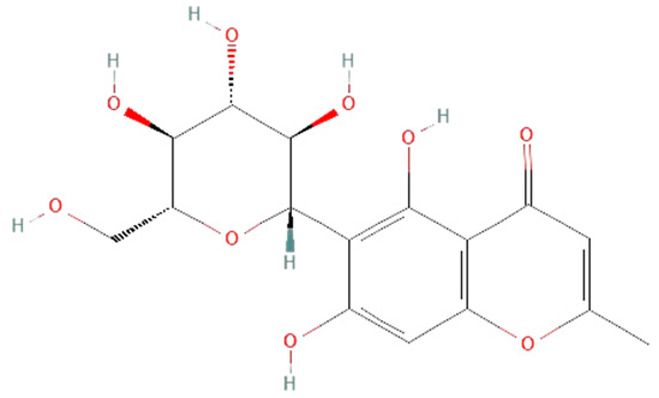		
*Zingiber officinale*	6-gingerol	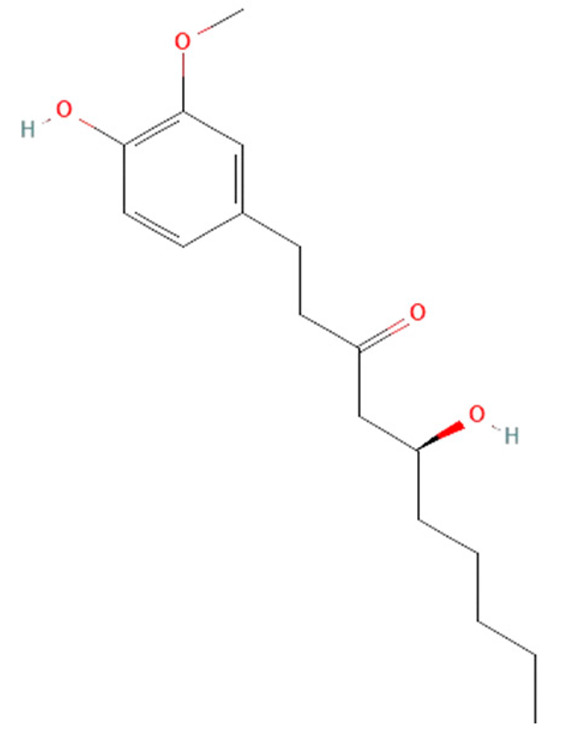	reduce levels of ROS, HMGB1, TNF-α, IL-6, IL-1β, and IL-10	[149,175,176,177,178,179]
	zingerone	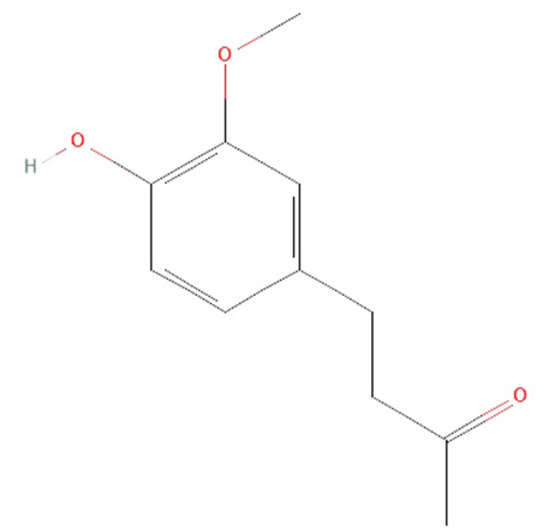		

iNOS—inducible nitric oxide synthases; NO•—nitric oxide; IL-6—interleukin 6; TNF-α—tumor necrosis factor alpha; ERK1/2—extracellular signal-regulated kinase 1/2; SOD—superoxide dismutase; GSH-Px—glutathione peroxidase; cAMP—cyclic adenosine monophosphate; TLR4—toll-like receptors 4; NF-kB—nuclear factor-kappa B; IL-1β—interleukin 1 beta; STAT1—signal transducers and activators of transcription 1; ROS—reactive oxygen species; HMGB1—high mobility group box 1; IFN—interferon; NOXs—nicotinamide adenine dinucleotide phosphate (NADPH) oxidases; IL-10—interleukin 10.

## Data Availability

No new data were created or analyzed in this study. Data sharing does not apply to this article. This review compiles the available information and collects data on sepsis research.
